# Sinomenine treats rheumatoid arthritis by inhibiting MMP9 and inflammatory cytokines expression: bioinformatics analysis and experimental validation

**DOI:** 10.1038/s41598-024-61769-x

**Published:** 2024-06-04

**Authors:** Jinfang Luo, Yi Zhu, Yang Yu, Yujie Chen, Kang He, Jianxin Liu

**Affiliations:** 1 Department of Basic Medicine, Department of Pharmacy, Guizhou University of Traditional Chinese Medicine, Guiyang, 550025 People’s Republic of China; 2 School of Pharmaceutical Sciences, Hunan University of Medicine, Jinxi South Road, Huaihua, 418000 People’s Republic of China; 3 College of Clinical Medicine, The Affiliated Zhongshan Hospital of Dalian University, Dalian, 116622 People’s Republic of China

**Keywords:** Rheumatoid arthritis, Bioinformatics, Sinomenine, M1 macrophages, MMP9, IL-6, TNF-α, Computational biology and bioinformatics, Immunology, Biomarkers, Rheumatology

## Abstract

Rheumatoid arthritis (RA) is a chronic systemic autoimmune disease marked by inflammatory cell infiltration and joint damage. The Chinese government has approved the prescription medication sinomenine (SIN), an effective anti-inflammation drug, for treating RA. This study evaluated the possible anti-inflammatory actions of SIN in RA based on bioinformatics analysis and experiments. Six microarray datasets were acquired from the gene expression omnibus (GEO) database. We used R software to identify differentially expressed genes (DEGs) and perform function evaluations. The CIBERSORT was used to calculate the abundance of 22 infiltrating immune cells. The weighted gene co-expression network analysis (WGCNA) was used to discover genes associated with M1 macrophages. Four public datasets were used to predict the genes of SIN. Following that, function enrichment analysis for hub genes was performed. The cytoHubba and least absolute shrinkage and selection operator (LASSO) were employed to select hub genes, and their diagnostic effectiveness was predicted using the receiver operator characteristic (ROC) curve. Molecular docking was undertaken to confirm the affinity between the SIN and hub gene. Furthermore, the therapeutic efficacy of SIN was validated in LPS-induced RAW264.7 cells line using Western blot and Enzyme-linked immunosorbent assay (ELISA). The matrix metalloproteinase 9 (MMP9) was identified as the hub M1 macrophages-related biomarker in RA using bioinformatic analysis and molecular docking. Our study indicated that MMP9 took part in IL-17 and TNF signaling pathways. Furthermore, we found that SIN suppresses the MMP9 protein overexpression and pro-inflammatory cytokines, including tumor necrosis factor-α (TNF-α) and interleukin-6 (IL-6) in the LPS-induced RAW264.7 cell line. In conclusion, our work sheds new light on the pathophysiology of RA and identifies *MMP9* as a possible RA key gene. In conclusion, the above findings demonstrate that SIN, from an emerging research perspective, might be a potential cost-effective anti-inflammatory medication for treating RA.

## Introduction

Rheumatoid arthritis (RA) is a chronic inflammatory joint disease commonly affecting the hands and feet. It is found in 0.5–1.0% of the worldwide population and is most common in women between 40 to 60 years old, with a prevalence of 0.5–2%^1–3^. It causes symptoms by deteriorating symmetrical polyarthritis that varies in extent and severity, resulting in irreparable cartilage and bone destruction^1–3^. Chronic synovitis of the affected joint is the most prevalent clinical symptom in RA patients, distinguished by persistent synovitis, synovial hyperplasia, and pannus formation, all of which deteriorate bone structure and eventually restrict joint function^4,5^. The underlying mechanism of RA is complex and associated with various factors, including genetics, the environment, and the immune system. Notably, the immune system plays a vital part in the overall progression of the disease, particularly during its initial phases^6,7^. Inflammation is a double-edged sword. While it predominantly serves as a protective response for the clearance and repair of injured tissues, its dysregulation may lead to chronic inflammation^8^. The abnormal immunity system is currently scientifically proven to have an important part in the progression of RA^7,9^. As a result, RA is now recognized as a chronic immune-mediated illness in which several immune cell types and communication networks malfunction, resulting in a maladaptive tissue repair process that causes organ damage, particularly in the joints^10^.

The immunohistological investigations and examinations of dissociated synovium revealed that macrophages, immune cells that participate in both the onset and resolution of inflammation, are the primary tumor necrosis factor-alpha (TNF-α) producing cells in the inflamed RA joint. Also, the therapeutic effectiveness of TNF inhibitors in this condition further demonstrates their relevance^11^. Cytokines have long been examined and studied as possible RA targets since they are directly implicated in the RA process. Cytokines can be classed as pro- and anti-inflammatory cytokines based on their antigen response roles. Macrophages produce cytokines that promote inflammation and lead to cartilage and bone damage^12^. Extended macrophage activation results in a dysfunctional inflammatory reaction involving the production of several inflammatory cytokines and mediators (including: IL-1, IL-6, IL-8, and TNF-α), leading to a vicious cycle of chronic inflammation^13^. Among these RA-related cytokines, the expression of TNF-α and IL-6 has been associated with the severity of joint pain^14^. Thus, reducing pro-inflammatory mediators may be a useful approach to treat and avoid chronic inflammatory diseases^15^.

Sinomenine (SIN, 7,8-dihydro-4hydroxy-3,7-dimethoxy-17-methyl-,13,14-morphinan-6-one) is an alkaloid isolated from Chinese medicinal *Caulis Sinomenii*. It had been utilized as a folk treatment in various regions of the Far East to cure neuralgia and rheumatic disorders in the 1920s^16^. Due to its high safety profile and potent anti-inflammatory and immune-regulatory effects, SIN has been widely investigated^17–20^. Previous research discovered that SIN demonstrated better clinical efficacy and fewer adverse events (including allergic reactions and gastrointestinal disorders) than methotrexate (MTX) therapy (a type of disease-modifying anti-rheumatic drug used to reduce the activity of the immune system) during RA treatment in the clinical setting^21^. SIN has been demonstrated to decrease the levels of inflammatory factors (TNF-α, IL-6, etc.) in IL-1β-induced RA-fibroblast-like synoviocytes (FLS), as well as the expression of toll-like receptor 4 (TLR4), myeloid Differentiation Primary Response Protein 88 (MyD88), p-NF-kB p65, and tumor necrosis factor receptor-associated factors 6 (TRAF-6)^22,23^. In cell or animal models of arthritis, the anti-inflammatory activity of SIN has been linked to RA development.

**Figure 1:**
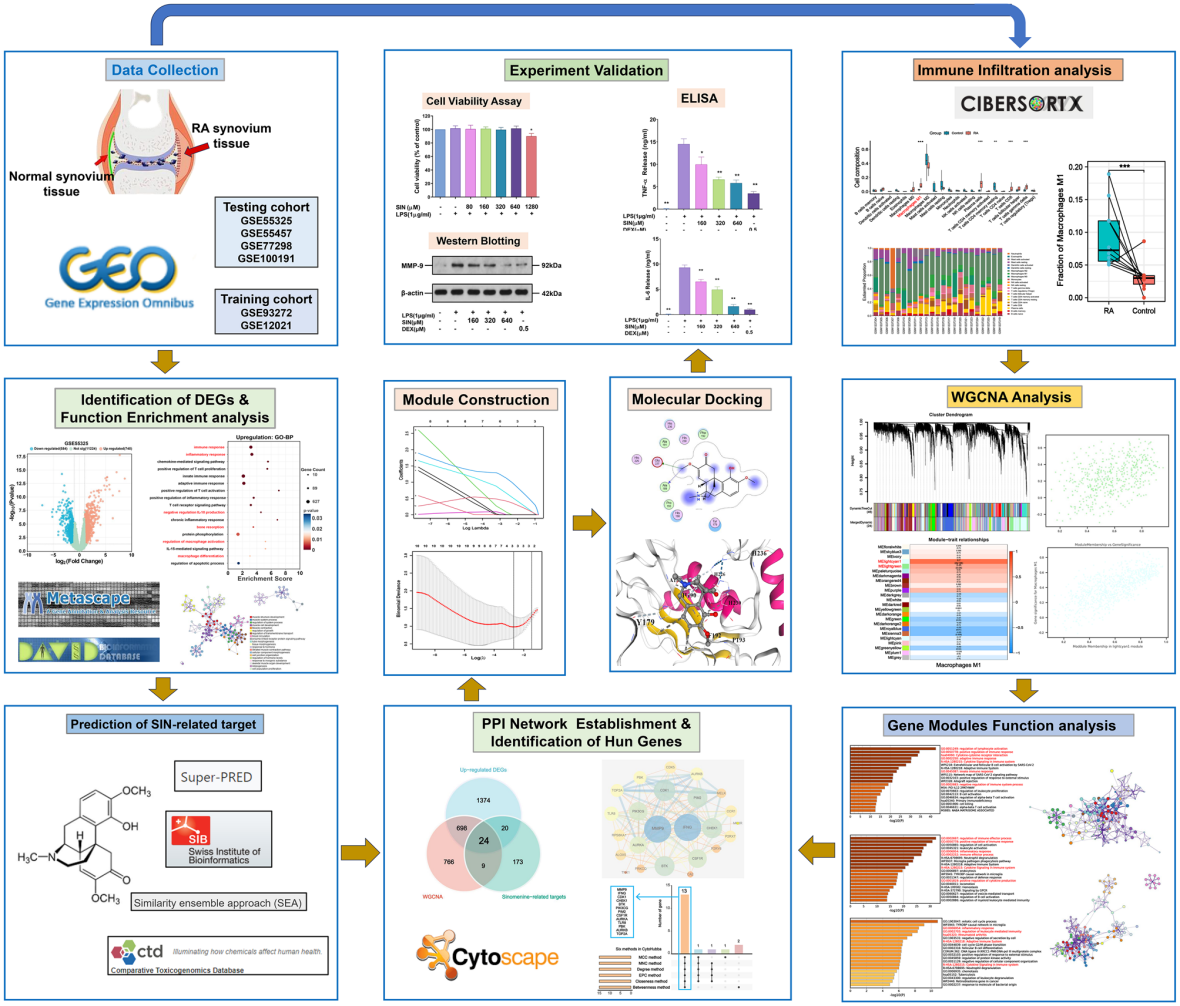
The flow diagram of this study. *DEGs* differentially expressed genes, *PPI* protein–protein interaction, *SIN* Sinomenine, *WGCNA* weighted co-expression network analysis, *GEO* gene expression omnibus.

SIN is already approved by Chinese government for RA treatment. However, the exact mechanism of the its action is not fully understood, which restricts the promotion and application worldwide to some extent. Thus, there is a need for using different approaches to explain the mechanisms of SIN. However, there is a lack of systematic bioinformatics analysis methods combined with experiments to verify its molecular mechanism. This work employed a variety of bioinformatics studies combined with experiments to verify its molecular mechanism for the first time. Briefly, GEO database was searched for GSE12021, GSE55325, GSE55457, and GSE77298 to discover DEGs between RA and AS. Then, CIBERSORT was used to examine infiltrating immune cells in RA utilizing testing and training microarray datasets. WGCNA was used to identify key module genes, after which we performed functional enrichment analysis. After screening DEGs and M1 macrophages-related genes, we used four public pharmacological target prediction databases to find SIN-related targets. We established a PPI network for the intersection genes and determined candidate genes with cytoHubba and the LASSO. The ROC curve was additionally employed for assessing biomarker predictiveness. The affinity of SIN and matrix metalloproteinases 9 (MMP9) protein was investigated using molecular docking. The impact of SIN on the expression level of MMP9 in RAW264.7 cells line stimulated with lipopolysaccharide (LPS) was assessed using Western blot. Additionally, we assessed the effects of SIN on the level of TNF-α and IL-6 in LPS-induced RAW264.7 cells using enzyme-linked immunosorbent assay (ELISA). These two cytokines have a crucial role in the progression of RA-related inflammation. In summary, we created a supplementary therapeutic strategy for RA by elucidating the mechanism of SIN in the treatment of RA, and expanded perspective. The flow chart of the study is presented in Fig. 1.

## Materials and methods

### Dataset collection and preprocessing

We retrieved 6 gene expression datasets from the Gene Expression Omnibus (GEO) (https://www.ncbi.nlm.nih.gov/geo/) database. The specific information of all data is presented in Table 1. Data were download from the GEO database using the “GEOquery” R package, missing values were filled with the “impute” R package, after which the data were normalized employing the “normalizebetweenarrays” R package. Eventually, differently expressed genes (DEGs) between RA and the control synovial tissues were obtained by applying p < 0.05 and |log_2_Fold change (FC)|> 1 in testing cohorts using the “limma” R package. The DEGs were visualized via the Sangerbox platform (http://vip.sangerbox.com/). The predictive targets for SIN were obtained using SwissTargetPrediction (http://www.swisstargetprediction.ch/), Similarity Ensemble Approach (SEA, http://sea.bkslab.org/), SuperPred (https://prediction.charite.de/) databases, and the comparative toxicogenomics database (CTD, https://ctdbase.org/).

**Table 1:** The information of six microarray datasets.

Dataset ID	Platform	RA		Control		Data type
		Type	Number	Type	Number	
GSE55325	GPL96	Synovium	10	Synovium	12	Testing
GSE55457	GPL96	Synovium	13	Synovium	10	Testing
GSE77298	GPL570	Synovium	16	Synovium	7	Testing
GSE100191	GPL13497	Whole blood	7	Whole blood	12	Testing
GSE93272	GPL570	Whole blood	232	Whole blood	43	Training
GSE12021	GPL96	Synovium	12	Synovium	9	Training

### Immune infiltration analyses via CIBERSORT algorithm

The online analytic tool CIBERSORTx (https://cibersortx.stanford.edu/) online tool^24^ was used to define the M1 macrophages infiltration status of RA and control synovial tissues in testing and training cohorts (except for the GSE93272 dataset), utilizing the default signature gene file (LM22, 22 kinds of immune cells) and 500 permutations. The results of the present study were filtered based on a p < 0.05, and each sample's M1 macrophages infiltration level was shown in a bar plot using the Wilcoxon test.

### Identifying the immune cell infiltration-related module via WGCNA

The weighted gene co-expression network analysis (WGCNA) method in OECloud (https://cloud.oebiotech.com) tool was used to determine the association between genes and infiltrating M1 macrophages abundance by constructing a gene co-expression network via using all genes in the testing and training cohorts. The Pearson correlation coefficient was used to compute the distance between each gene, and the WGCNA program was used to create the correlation adjacency matrix. The “hclust” function was used to do the hierarchical clustering analysis, and the “pickSoftThreshold” function was used to get the soft thresholding power value. In addition, the corresponding modules were built via the “blockwiseModules” function. Both module membership (MM) and gene significance (GS) score ≥ 0.5 were used to identify the hub immune genes in the most relevant modules.

### Function enrichment determination

Gene ontology (GO) and Kyoto encyclopedia of genes and genomes (KEGG) pathway enrichment analyses were performed with the use of the database for annotation, visualization, and integrated discovery (DAVID, https://david.ncifcrf.gov/) database for the function and pathway of the identified gene^25^, Enrichr (http://amp.pharm.mssm.edu/Enrichr) database^26^, and Metascape (https://metascape.org) public database to undertake functional annotation analysis of hub genes^27^. A p < 0.05 represented the statistical significance.

### Protein–protein intersection (PPI) network construction and screening of hub genes

The common upregulated DEGs, SIN-related genes, and the M1 macrophages-related genes were obtained using the Venn diagram. Then, the protein–protein intersection (PPI) network of overlapping DEGs was analyzed using the STRING (https://string-db.org) database with reliable filtering criteria (score > 0.1), and the network was further analyzed by employing Cytoscape (version: 3.7.1) software. The cytoHubba plugin in the Cytoscape (version: 3.9.1) software was used to score each node gene using seven algorithms, namely MCC (maximal clique centrality), MNC (maximum neighborhood component), Degree, EPC (edge percolated component), closeness, and betweenness. The “UpSet” R program screened hub genes using the top 15 node genes scored by each method.

### Construction and validation of the prognostic value of hub genes

Least absolute shrinkage and selection operator (LASSO) method was used to further study possible candidate genes for identifying RA patients. Predictive accuracy may be improved by using LASSO, a machine-learning approach that combines variable selection and regularization^24,28^. To verify the selected diagnostic genes, we used the testing and testing cohort. The diagnostic effectiveness of diagnostic markers was assessed using a receiver operating characteristic (ROC) curve. An area under curve (AUC) > 0.9 indicated that the gene had a good fitting effect.

### Molecular docking stimulation analysis

Molecular docking analysis was performed with the protein targets of the chosen ligands using CB-Dock2 (https://cadd.labshare.cn/cb-dock2/php/index.php) platform^29^. CB-Dock2 is a molecular docking tool based on AutoDock Vina analysis which can automatically identify and analyze the site where the ligand binds to the receptor. It enhances molecular docking accuracy while simplifying docking processes. The SIN structure was imported into the CB-Dock2 server by obtaining it from the PubChem database (https://pubchem.ncbi.nlm.nih.gov). After processing the provided SIN, partial charges and hydrogens will be added, and RDKit will produce an initial 3D conformation. The RCSB protein data bank (RCSB PDB, https://www.rcsb.org) database was employed for obtaining the 3D structure of MMP9 crystals (Protein ID: 4XCT). The submitted MMP9 will be checked by CB-Dock2, which will also add the missing side-chain^30,31^ and hydrogen atoms, notify about any missing residues in a protein, remove co-crystallized waters and other het groups, and deliver alerts about any missing residues^32^. We made assured that the whole SIN was contained within the grid box. The Vina score was computed using CB-Dock2, which projected the optimal docking model with the least amount of energy. The “Cartoon” option was selected for the ligand and receptor. The terms “Element” and “Secondary structure” were used to present ligands and colored receptors, respectively. The following screening criteria were used to find important active ingredients: (1) compounds with the greatest affinity for each crucial therapeutic target; (2) compounds with a docking energy of < − 5 kcal/mol that operates on the principal therapeutic targets. Finally, using the Molecular Operating Environment (MOE version: 2020.02) software, the details of ligand-receptor interactions were revealed in the receptor-ligand interactions window.

### Experiments validation

#### Materials

Sinomenine (SIN) (purity > 99%) was purchased from Chengdu Si Ke Hua biological technology Co. Lipopolysaccharide (LPS) and dexamethasone (DEX) were purchased from Sigma (St. Louis, MO, USA). RAW264.7 cell line was obtained from American Type Culture Collection (ATCC, Manassas, VA, USA). CCK8 was from TargetMol. Primary antibody to MMP9 and β-actin purchased from Wuhan Sanying Biotechnology Co., LTD. ELISA kit for TNF-α and IL-6 ELISA Kit purchased from MultiSciences Biotech Co., LTD. The secondary antibody (Anti-rabbit or Anti-mouse) was purchased from Wuhan Doctor De Biological Engineering Co., LTD.

#### Cells cultures and treatment

RAW264.7 cells were cultured in Dulbecco's Modified Eagle Medium (DMEM) supplemented with 10% fetal bovine serum (FBS) and 1% penicillin/streptomycin in a humidified atmosphere containing 5%CO_2_/95% air at 37 °C. Cells were sub-cultured 3 times before subsequent experiments.

#### Cell viability assay

Cell activity was detected by using a CCK8 kit. A total of 1 × 10^5^ cells/ml were seeded in 96-well plates (100 µL per well) and incubated overnight at 37 °C. Cells were then exposed to gradually increased concentrations of SIN (80, 260, 320, 640, and 1280 µM) or DEX (0.125, 0.25, 0.5 µM) for 1 h and then with LPS for another 18 h at 37 °C. Next, 10 µL of sterile CCK 8 solution was added to each well and incubated for another 2 h at 37 °C. The absorbance at 450 nm was determined using a microplate reader.

#### ELISA assay

Inflammatory factors (TNF-α and IL-6) were detected by ELISA kit. RAW 264.7 cells were adjusted cell density to 3 × 10^5^ cells/mL (2 mL/well) into a 6-well culture plate overnight. Then, cells were treated with or without drugs (SIN or DEX) for 1 h and with LPS for another 18 h. After incubation, the supernatant solution was collected, centrifuged for 10 min (1200 rpm), and the content of TNF-α and IL-6 in the cell supernatant was detected using the ELISA kit. In brief, the samples were added to 96-well plates (n = 3), the primary antibody (for detecting TNF-α and IL-6) was added, incubated for 1 h, washed the plate, then the secondary antibody was added, incubated for 0.5 h, the chromogenic solution accompanying the kit was added, and the OD value was measured at 450 nm according to the instructions of the kit.

#### Western blot assay

The RAW264.7 cells line was seeded into 6-well plates (1.5 × 10^6^ per well), and the cells were treated with SIN or DEX for 1 h and then with LPS (1 µg/mL) for 18 h. The cells were lysed with radio immunoprecipitation assay lysis buffer (RIPA) lysis buffer that contained protease inhibitors, and the concentration of proteins was then detected using the bicinchoninic acid assay (BCA) kit. Next, the samples (30–40 µg) were separated by sodium dodecyl sulfate polyacrylamide gel electrophoresis (SDS-PAGE) and transferred to the polyvinylidene fluoride (PVDF) membrane. After being blocked in 1 × TBST with 5% bovine serum albumin (BSA) for 1 h at RT, the membranes were incubated with primary antibodies, including MMP9 (1:1000) and β-actin (1:2000) overnight (4 °C). The membranes were then washed three times using 1 × TBST and incubated with appropriate anti-rabbit IgG (1:3000) or anti-mouse IgG (1:3000) secondary antibody for 2 h at room temperature (RT). After this, the levels of each protein were performed by enhanced chemiluminescence (ECL, Millipore). ImageJ software was used to calculate the relative density of the proteins.

### Statistical analysis

Data are the mean value ± standard deviation (SD). Wilcoxon tests were used to compare two groups. Three or more groups were compared by the One-Way ANOVA. Correlation analysis was assessed using Pearson correlation. A p < 0.05 had been considered statistically significant.

## Results

### Screening of DEGs in different datasets and enrichment analysis

**Figure 2:**
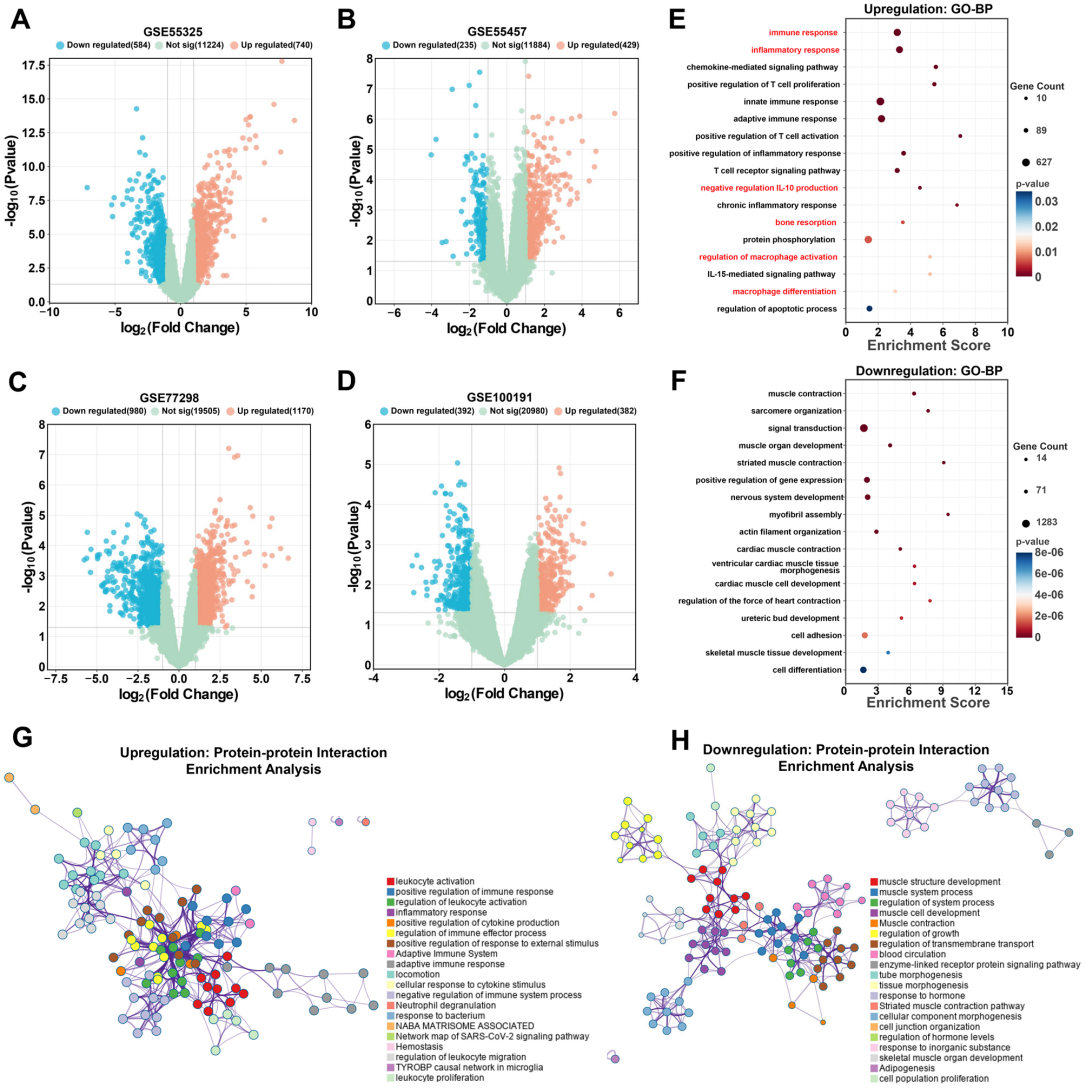
Data preprocessing and enrichment analysis. (**A**–**D**) Volcano graphs showing DEG distribution in the datasets GSE55325, GSE55457, GSE77298, and GSE100191. Upregulated DEGs are represented by orange dots, whereas downregulated DEGs are represented by blue dots; green dots represent genes that were not differentially expressed. (**E**,**F**) GO-BP examines the effects of the upregulation and downregulation of DEGs. (**G**,**H**) A visualization of the metascape protein–protein interactions enrichment network illustrating the intra-cluster and inter-cluster similarities of enriched phrases, with up to 10 terms per cluster. Cluster annotations are color coded.

In this study, four testing cohorts were employed for identifying DEGs with p < 0.05 and |log_2_Fold change (FC)|> 1. Using the GSE55325 dataset, an analysis of volcano plots discovered 1324 DEGs, consisting of 740 upregulated and 584 downregulated genes (Fig. 2A). Similarly, in the GSE55457 datasets, 664 DEGs were identified, of which 429 genes were upregulated and 235 were downregulated (Fig. 2B); In the GSE77298 datasets,150 DEGs were screened, with 1170 upregulated and 980 downregulated genes (Fig. 2C); In the GSE100191 datasets, 774 DEGs were found, with 382 upregulated and 392 downregulated genes (Fig. 2D). The enrichment analysis of the database for DAVID enrichment results revealed that upregulated DEGs were significantly related to the immune system, inflammatory process, and macrophages, namely immune response, inflammatory response, negative regulation of IL-10 production, bone resorption, regulation of macrophage activation, and IL-15-mediated signaling pathway (Fig. 2E), while downregulated DEGs were significantly enriched in signal transduction, positive regulation of gene expression, and cell adhesion from the results from the DAVID database (Fig. 2F). The enrichment analysis of Metascape results further revealed that upregulated DEGs were markedly associated with RA, including inflammatory response, regulation of immune effector process, and adaptive immune response (Fig. 2G). Meanwhile, downregulated DEGs were markedly enriched in muscle structure development, cellular component morphogenesis, and regulation of transmembrane transport (Fig. 2H).

### Infiltration of immune cells in RA and control synovial tissues

**Figure 3:**
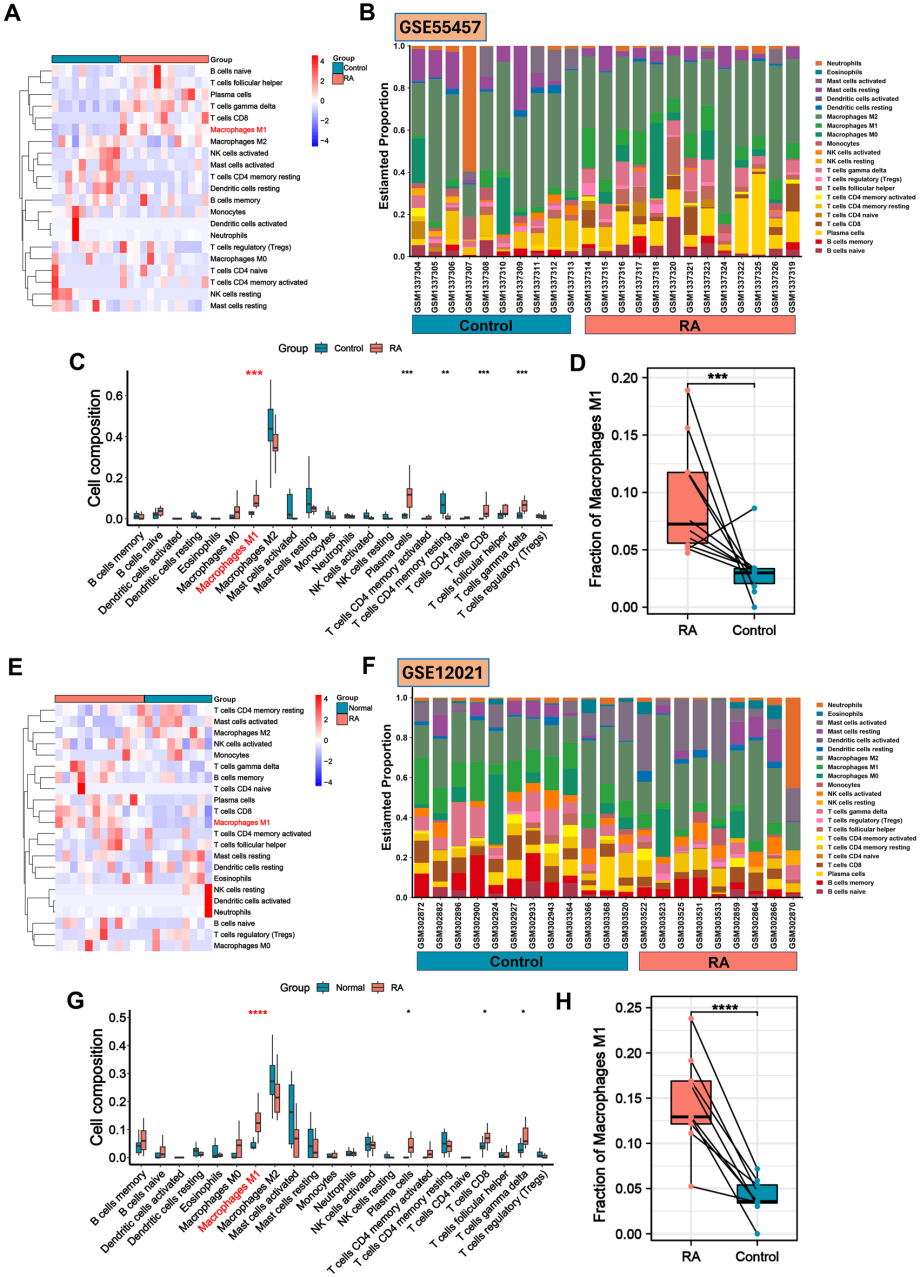
The landscape of 22 infiltrating immune cells in the GSE55457 and GSE12021 datasets. (**A**) A heat map depicting the abundance of 22 immune cell populations infiltrating in GSE55457 dataset. (**B**) In the fractions of 22 subsets of immune cells in RA and control samples in the GSE55457 dataset, different colors indicate different immune cells. Ten samples on the left are control subjects, and 13 on the right are patients with RA. (**C**) The boxplot shows the difference in immune infiltration between control and RA samples in the GSE55457 dataset; the control samples are in blue, and the RA samples are in red. (**D**) The boxplot shows the difference in M1 macrophages between control and RA samples in the GSE55457 dataset. (**E**) A heat map depicting the abundance of 22 immune cell populations infiltrating the GSE12021 dataset. (**F**) In the fractions of 22 subsets of immune cells in RA and control samples in the GSE12021 dataset, different colors indicate different immune cells. 12 samples on the left are control subjects, and 9 on the right are patients with RA. (**G**) The boxplot shows the difference in immune infiltration between control and RA samples in the GSE12021 dataset; the control samples are in blue, and the RA samples are in red. (**H**) The boxplot shows the difference in M1 macrophages between control and RA samples in the GSE12021 dataset. Statistical level: *p < 0.05; **p < 0.01; ***p < 0.001, and ****p < 0.0001.

Considering the essential diagnostic genes associated with RA are primarily immune-related, immune infiltration analysis was used to investigate the role of immunity in RA. In this study, the CIBERSORT method was utilized to assess the relative distribution of 22 subgroups of immune cells in GSE55457 and GSE12021 (other datasets did not yield significant results). The Wilcoxon test was used to determine the significantly different infiltrating immune cells in RA and control synovial tissues. For the GSE55457 dataset, 13 RA and 10 control synovial samples were calculated via the CIBERSORT algorithm. Initially, a heat map (Fig. 3A) and a histogram (Fig. 3B) were constructed to indicate the makeup of each sample's 22 distinct kinds of immune cells. The histogram displays the percentage of distinct immune cells in every sample, amounting to 1. Meanwhile, the heat map illustrates each sample's normalized absolute abundance of immune cells. We discovered that M1 macrophages, naïve B cells, plasma cells, CD8 + T cells, and γδ T cells were the main infiltrating immune cells in RA. As shown in the violin plot of the difference among 22 immune cell infiltrations (Fig. 3C), M1 macrophages, plasma cells, CD8 + T cells, and γδ T cells in RA samples presented a high infiltration compared with the control sample, while the resting memory CD4 + T cells were significantly infiltrated in control samples. Among these cells, M1 macrophages (p < 0.001) were selected for further analysis (Fig. 3D). Meanwhile, we also employed the same methodology on the GSE12021 dataset, which contains 12 RA and 9 control synovial samples. The results showed that M1 macrophages, plasma cells, CD8 + T cells, and γδ T cells were the main infiltrating immune cells in RA (Fig. 3E,F). As shown in the violin plot of the difference among 22 immune cell infiltrations (Fig. 3G), M1 macrophages, plasma cells, CD8 + T cells, and γδ T cells in RA samples presented a high infiltration compared with the control sample. Among these cells, M1 macrophages (p < 0.001) were selected for the next analysis (Fig. 3H).

### Identification of M1 macrophages-related genes modules and critical genes

**Figure 4:**
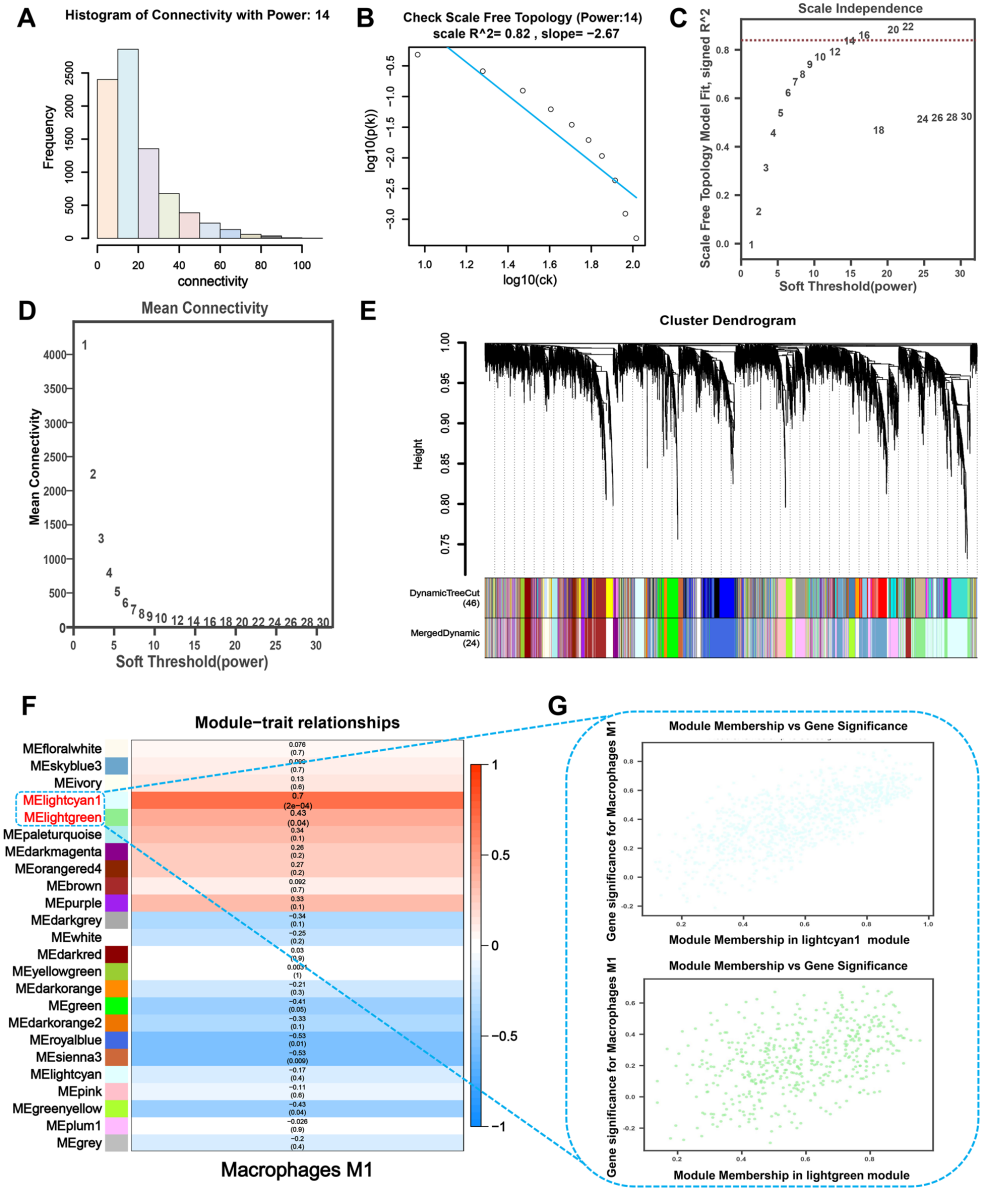
Gene co-expression networks were identified in the GSE55457 dataset using WGCNA, and potential biomarkers associated with M1 macrophages in RA were screened. (**A**) A histogram of the connection distribution when *β* = 14. (**B**) Checking the scale-free topology when *β* = 14. (**C**) A comparison of scale-free fitting indices for different soft-thresholding powers (*β*). (**D**) Analysis of mean connectivity of various soft-thresholding powers. (**E**) Hierarchical clustering of genes based on the 1-TOM matrix was used to organize the cluster dendrogram of the co-expression network modules. Different colors represent modules. The Dynamic Tree Cut colors reflect the modules detected by the dynamic Tree Cut approach, and because certain modules are linked, they are combined into the same module (combined Dynamic modules). (**F**) Significant variations were observed in both the gene module and the population of M1 macrophages when comparing samples from individuals with RA to those from healthy individuals. The color change in M1 macrophages indicates the correlation between the modules and the M1 macrophages. Additionally, the accompanying p-value reflects this correlation. (**G**) Scatter plots demonstrate the association between MElightcyan1 and MElightgreen module membership (MM) and gene significance (GS). Solid dots denote genes with MM ≥ 0.5 and GS ≥ 0.5.

To better comprehend the relationship between gene expression and the quantity of M1 macrophages, we built a WGCNA in the GSE55457 and GSE12021 datasets using the OECloud tool and the CIBERSORT method. For the GSE55457 dataset, the top 61% of variable genes (8141 genes) were included in the WGCNA. After selecting the power of *β* = 14 (scale-free network *R*^2^ = 0.82) as the soft threshold to ensure a scale-free network (Fig. 4A–D), 8141 genes were grouped into 24 modules, with the grey module, including genes that could not be assigned to any module (Fig. 4E). The module-trait relationships heat map between gene modules and the abundance of M1 macrophages populations that were significantly infiltrated in the RA samples compared to the control sample are shown in Fig. 4F. The MElightcyan1 (r = 0.7, p < 0.001) and MElightgreen (r = 0.43, p = 0.04) modules had the significantly highest correlation with the M1 macrophages. Eventually, 1422 M1 macrophage-related genes were identified from these two modules. Scatter plots show the GS and MM values for the MElightcyan1 and MElightgreen modules in the M1 macrophages populations, and genes with both MM and GS scores ≥ 0.5 were chosen as potential genes (Fig. 4G).

**Figure 5:**
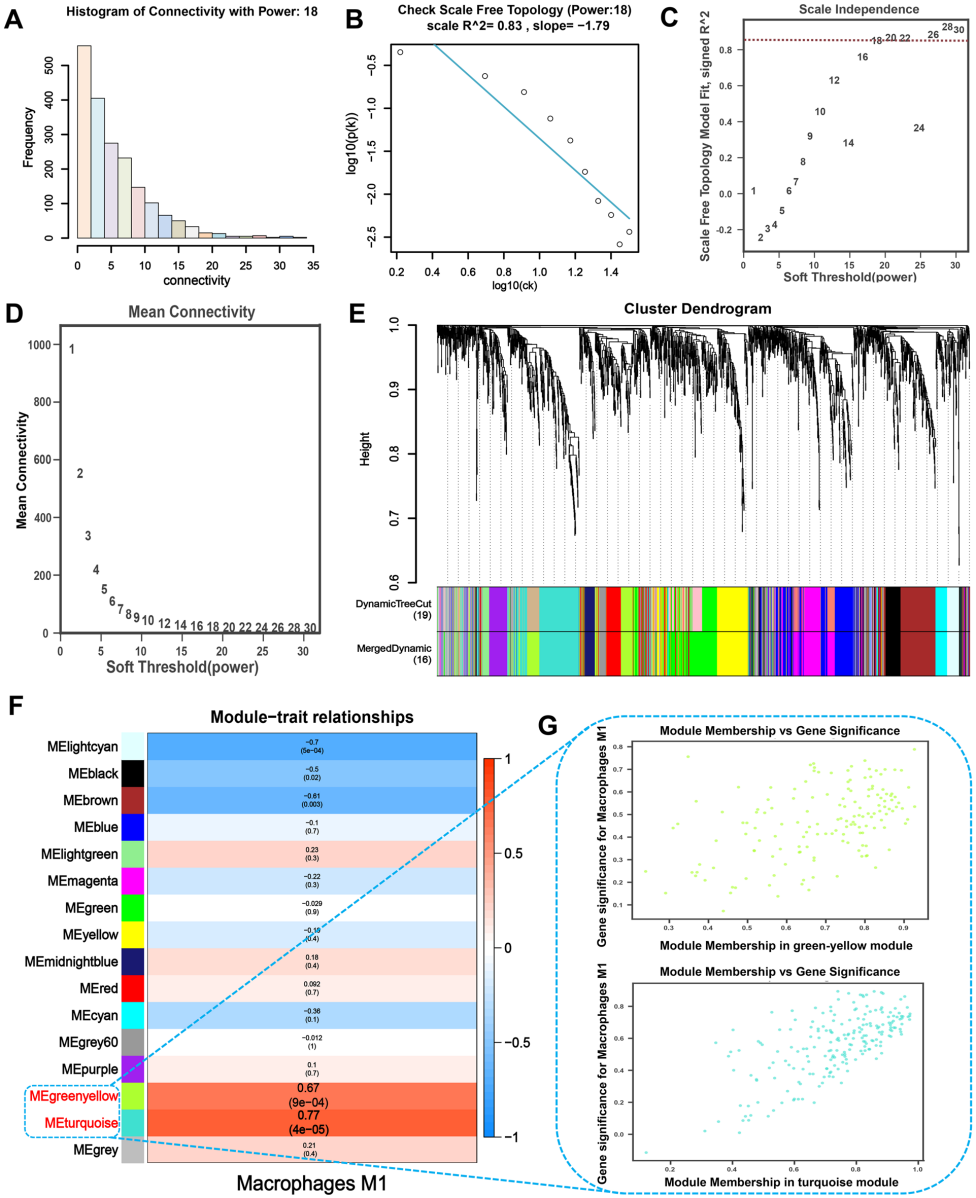
Gene co-expression networks were identified in the GSE12021 dataset using WGCNA, and potential biomarkers associated with M1 macrophages in RA were screened. (**A**) Histogram of the connection distribution when *β* = 18. (**B**) Checking the scale-free topology when *β* = 18. (**C**) The scale-free fitting indices for various soft-thresholding powers (*β*) are examined. (**D**) Analysis of mean connectivity of various soft-thresholding powers. (**E**) Hierarchical clustering of genes based on the 1-TOM matrix is used to organize the cluster dendrogram of the co-expression network modules. Different colors represent modules. The Dynamic Tree Cut colors represent the modules identified by the dynamic tree cut technique, and because certain modules have some correlation, the related modules will be merged into the same module (Merged Dynamic modules). (**F**) Correlation between the gene module and the M1 macrophages population, which differed considerably between the RA and healthy samples. In M1 macrophages, the correlation coefficient reflected the correlation between the gene module and the M1 macrophages, which changed color from red to blue. The associated p-value is also indicated. (**G**) Scatter plots depict the relationship between module membership (MM) and gene significance (GS) in the MEgreenyellow and MEturquoise module. Genes with MM ≥ 0.5 and GS ≥ 0.5 are labeled with solid dots.

Subsequently, the WGCNA includes the top 26% of variable genes (1921 genes) from the GSE12021 dataset. To achieve a scale-free network, we used a power of *β* = 18 (scale-free network *R*^2^ = 0.83) as the soft threshold (Fig. 5A–D). In addition, 1921 genes were retrieved for further study after being classified into 16 modules, with the grey module including genes that could not be allocated to any module (Fig. 5E). The heatmap of module-trait relationships demonstrates that in RA samples, gene modules and the amount of M1 macrophages were significantly enriched (Fig. 5F). The MEgreenyellow (r = 0.67, p < 0.001) and turquoise (r = 0.77, p < 0.0001) modules correlated the most with M1 macrophages. From these two modules, 357 M1 macrophages-related genes were discovered. Scatter plots show the GS and MM values for the MEgreenyellow and MEturquoise modules in the M1 macrophages populations, and genes with both MM and GS scores ≥ 0.5 were chosen as candidate genes (Fig. 5G).

### Functional enrichment analysis of key genes in M1 macrophages-related modules

**Figure 6:**
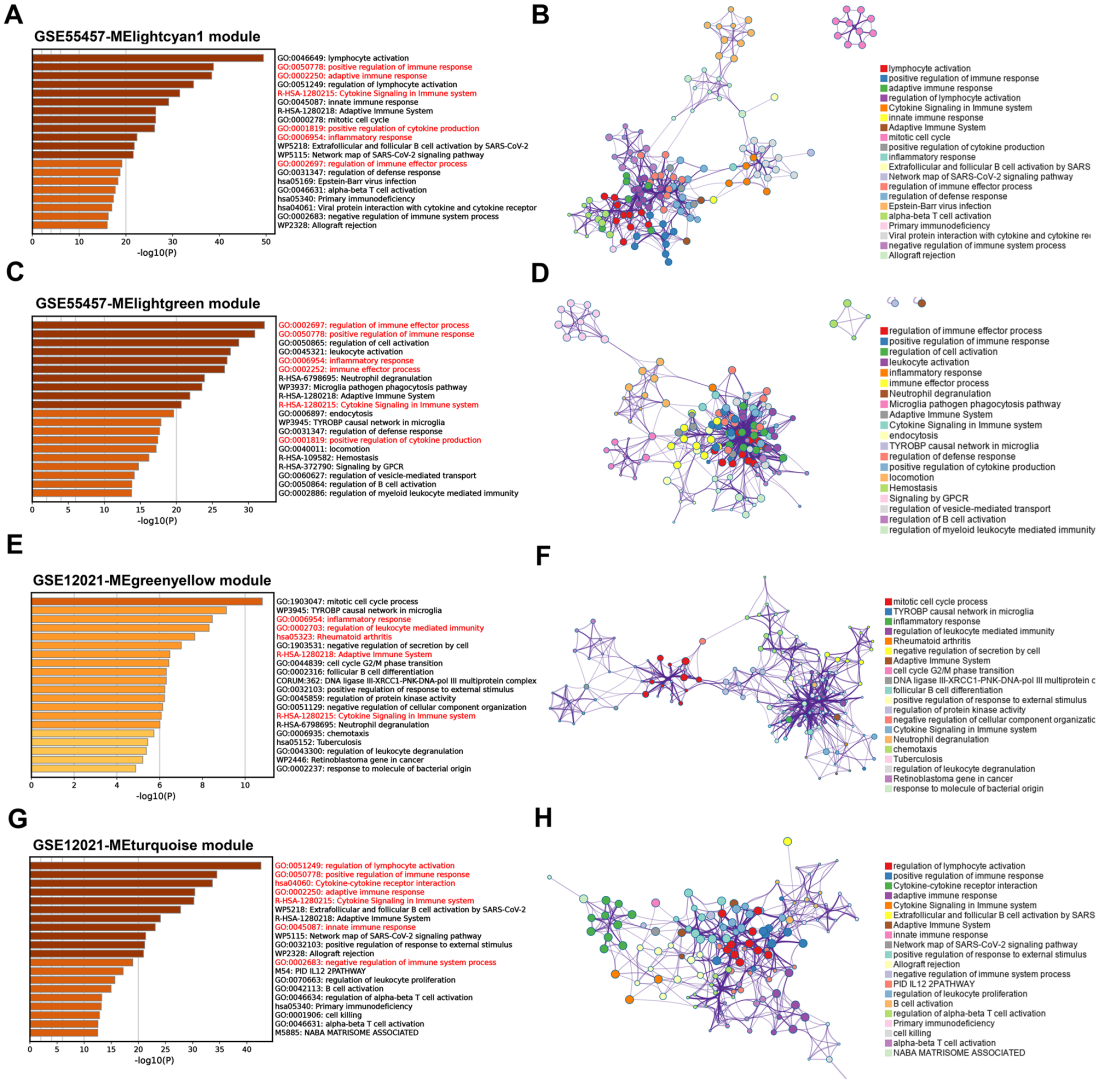
Functional enrichment analysis of four identified M1 macrophages-related modules of GSE55457 and GSE12021 datasets from Metascape. (**A**–**D**) The top 20 most significant pathways and GO terms of two identified M1 macrophages-related modules in the GSE55457 dataset. (**E**–**H**) The top 20 most significant pathways and GO terms of two identified M1 macrophages related modules in the GSE12021 dataset.

Employing Metascape, enrichment analysis of genes in M1 macrophages-related modules chosen from the GSE55457 and GSE12021 datasets was performed based on WGCNA. In the GSE55457 database, we identified two hub modules, namely MElightcyan1 (including 937 genes) and MElightgreen (including 485 genes). Genes in the MElightcyan1 module and MElightgreen were primarily associated with immune, inflammatory response, and cytokines production, notably positive regulation of immune response, positive regulation of cytokines production, and inflammatory response, all of which are remarkably associated with the development and progression of RA (Fig. 6A–D). Meanwhile, we also identified two critical modules in the GSE12021 dataset, including MEgreenyellow (including 151 genes) and MEturquoise (including 206 genes), finding that these genes were mainly enriched in inflammatory response, rheumatoid arthritis, cytokine signaling in the immune system, cytokine-cytokine receptor interaction, and negative regulation of immune system process, which suggested that the majority of them were involved in immunological and inflammatory activities (Fig. 6E–H). These findings suggest that immunological and inflammatory responses have essential roles in RA and may contribute to the disease progression.

### Enrichment analysis of hub genes and screening node genes via the PPI network

**Figure 7:**
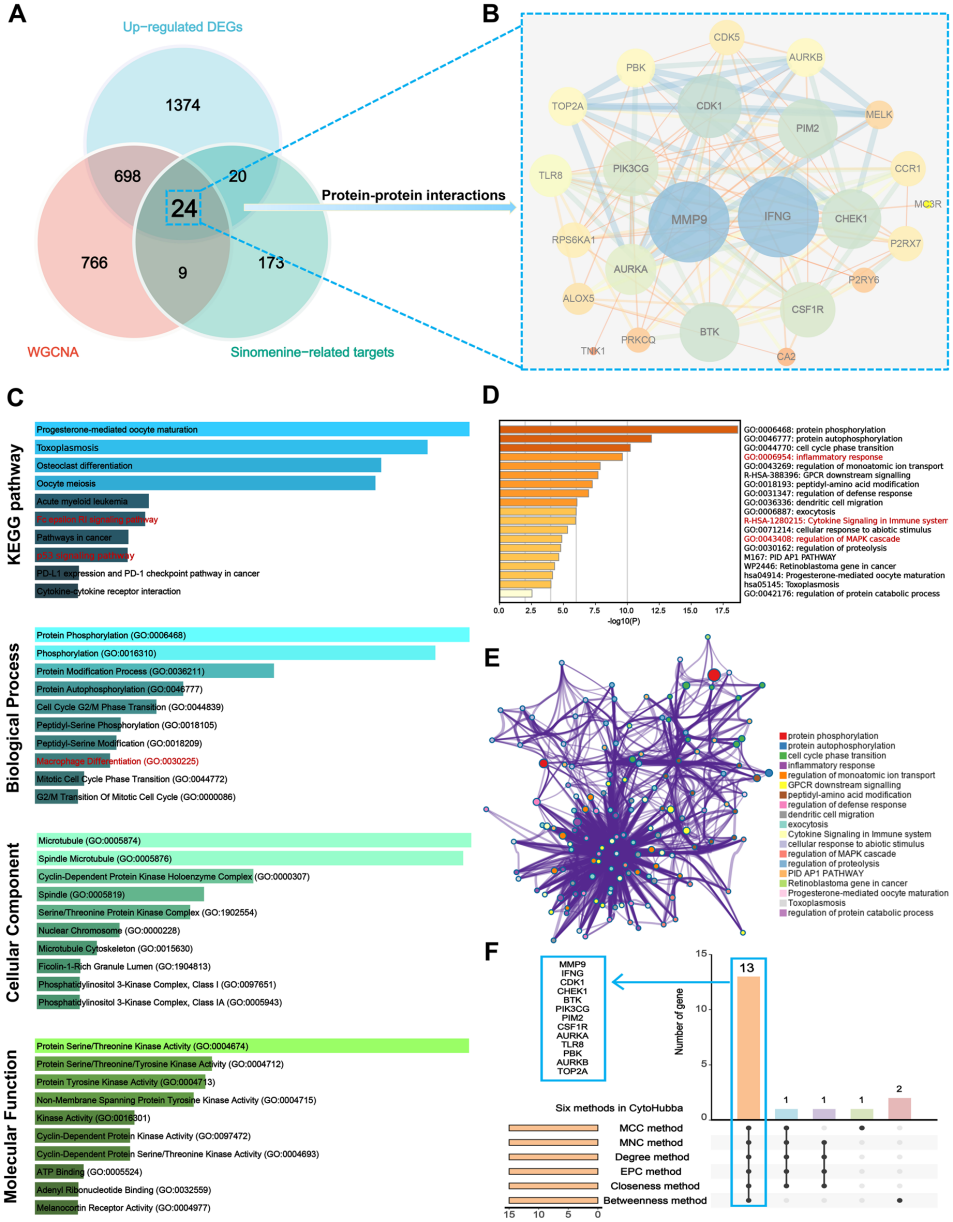
The PPI network for RA was used to perform function enrichment analysis of gene intersections and node gene recognition. (**A**) The intersection of the hub target genes tested by the three approaches was determined using a Venn diagram. (**B**) PPI network presents the relationship of 24 overlapped hub genes. (**C**) The results of KEGG and GO (biological process, cellular components, and molecular function) analysis were presented by bar plot. (**D**) The bar plot presents the results of KEGG and GO enrichment analysis from Metascape. (**E**) A visualization of the Metascape protein–protein interactions enrichment network illustrating the intra-cluster and inter-cluster similarities of enriched phrases, with up to 10 terms per cluster. Cluster annotations are color coded. (**F**) Six algorithms form cytoHubba software to screen hub genes by the upset plot.

Based on SwissTargetPrediction, SEA, SuperPred, and CTD databases, 226 SIN-related target genes were screened. Venn plot presents the 24 overlapping genes among the SIN-related target gene, upregulated DEGs, and M1 macrophages-related genes (Fig. 7A). We established a PPI network for 24 genes using the STRING database and analyzed it with Cytoscape3.7.0 software. Our analysis indicated *MMP9* and *interferon gamma (IFNG)* as potential significant targets for RA (Fig. 7B). Enrichment analysis was performed on these 24 overlapping genes to investigate the association between RA-related genes and the development of RA. The KEGG and GO analysis from the Enrichr database showed that 24 genes mainly enriched in the Fc epsilon RI signaling pathway, cytokine-cytokine receptor interaction, macrophage differentiation, cyclin-dependent protein kinase complex, and protein serine/threonine kinase activity, which were all closely associated with the immune system (Fig. 7C). Next, Metascape was used to run GO and KEGG enrichment analyses for further investigation. Results showed that they mainly participated in the inflammatory response and cytokine signaling in the immune system, indicating that most were involved in immune and inflammatory-related functions (Fig. 7D,E). Finally, a total of 13 core genes, including *MMP9, IFNG, CDK1, CHEK1, BTK, PIK3CG, PIM2, CSF1R, AURKA, TLR8, PBK, AURKB,* and *TOP2A,* were identified based on the overlapped parameters of the top 15 hub genes in 6 algorithms (Fig. 7F).

### Screening and verification of diagnostic genes in RA patients

**Figure 8:**
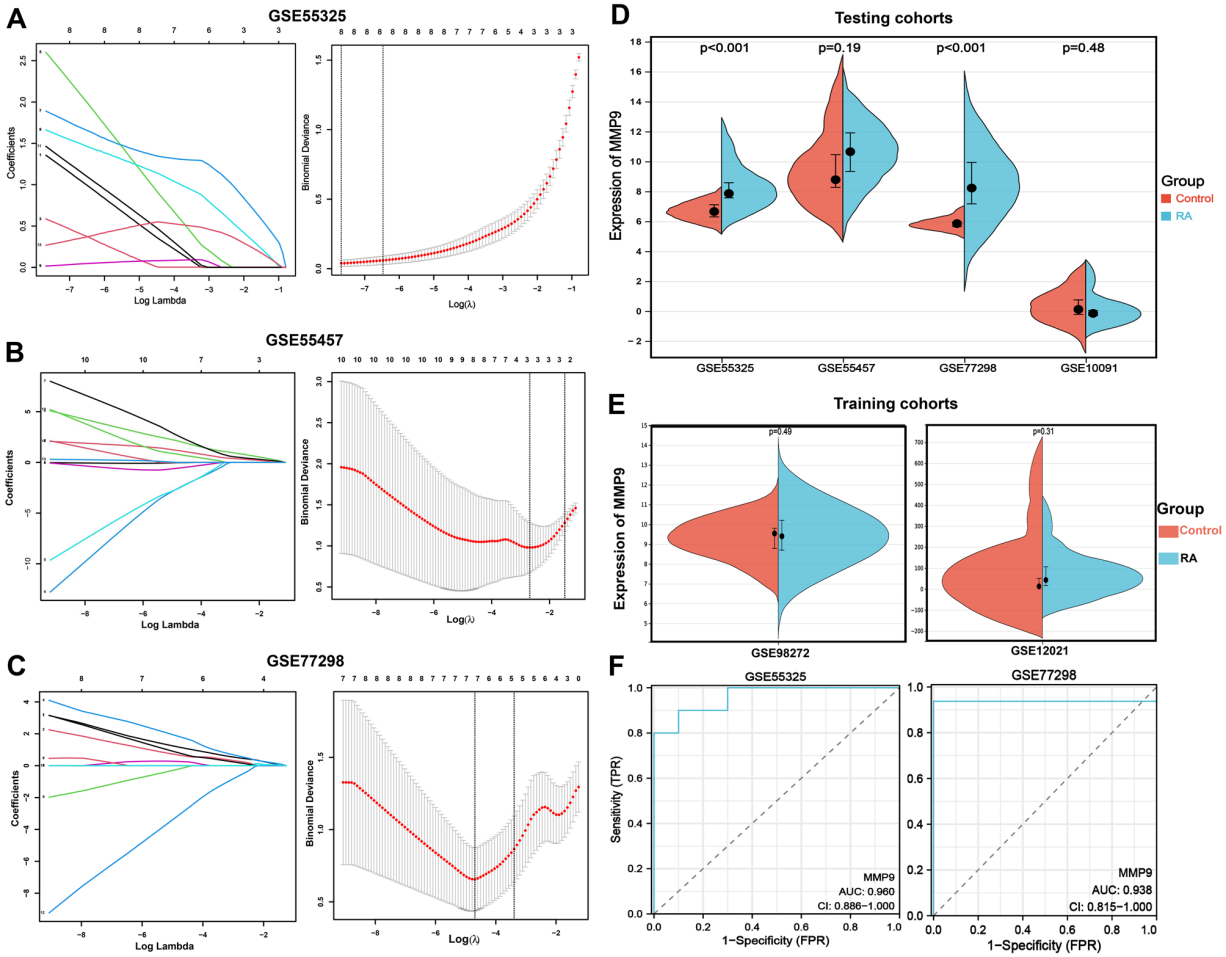
Screening for diagnostic markers in RA using a comprehensive approach. (**A**–**C**) The logistic regression approach of LASSO is used to determine diagnostic biomarkers, and various colors represent distinct genes. (**D**) Expression difference of the *MMP9* between RA and control groups in the testing cohorts. (**E**) Expression difference of the *MMP9* between RA and control groups in the training cohorts. (**F**) The ROC curve of *MMP9* between RA and control groups in the GSE55325 and GSE77298 datasets.

**Figure 9:**
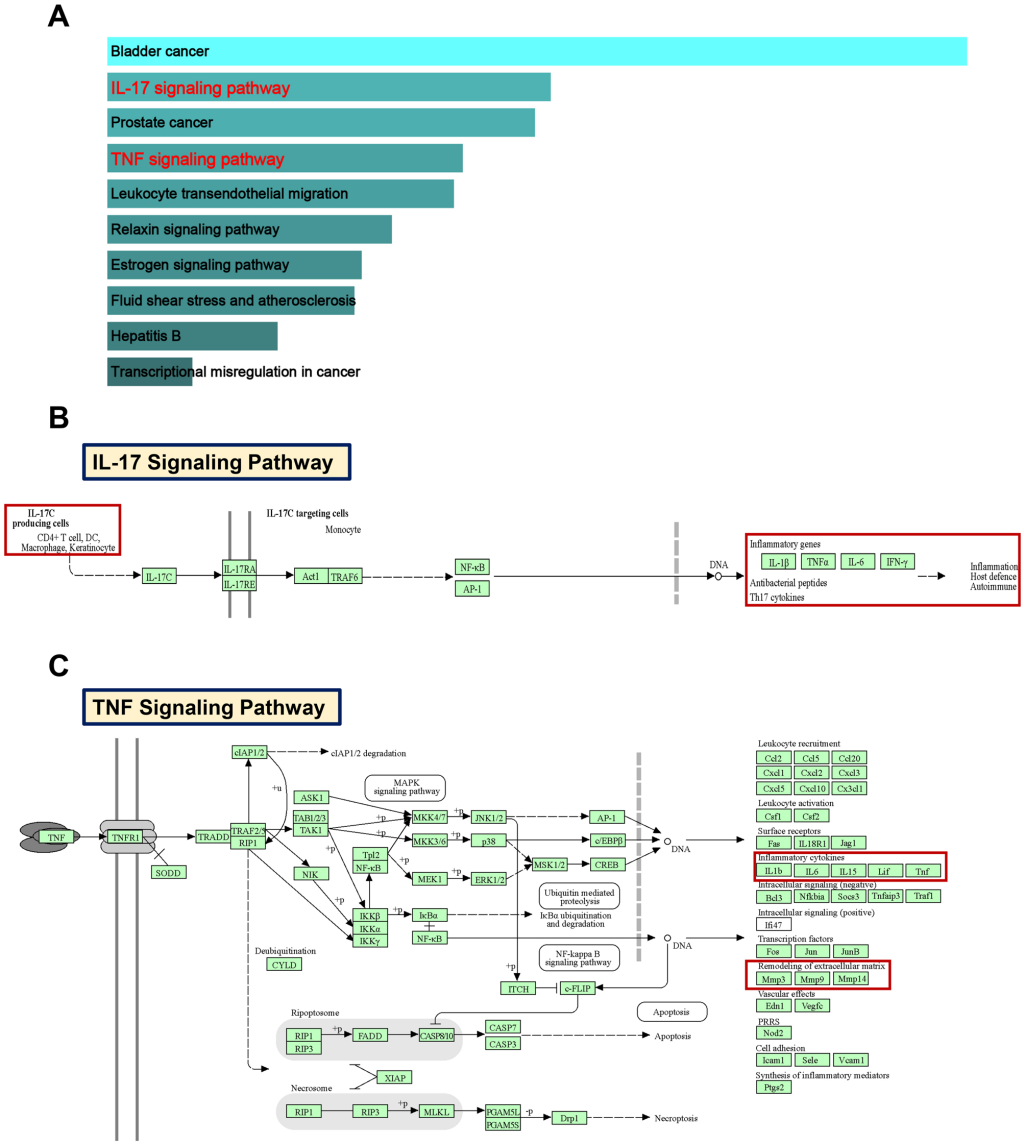
The IL-17 and TNF signaling pathway was significantly enriched in the RA. (**A**) KEGG analysis is presented by bar plot. (**B**,**C**) The pro-inflammatory cytokines or proteins, namely IL-6, TNF-α, and MMP9, participate in the IL-17 signaling pathway and TNF signaling pathway from the KEGG database.

For a better understanding of the diagnostic potential of hub genes, we then constructed a prediction model for the diagnosis of RA using the LASSO algorithm to distinguish the RA patients from healthy controls in the testing cohorts (*MMP9* in the GSE100191 dataset was not significant with zero coefficient). *MMP9* was screened with non-zero coefficients in GSE55325, GSE55457, and GSE77298 datasets (Fig. 8A–C). In the testing set, *MMP9* was significantly overexpressed in RA compared with the control in the GSE55325 (p < 0.001) and GSE77298 (p < 0.001). However, *MMP9* was not significant in RA compared with the control in the GSE55457 and GSE10091 datasets (Fig. 8D). In the training cohorts, *MMP9* was not significant in GSE98272 and GSE12021 datasets (Fig. 8E). Additionally, ROC analysis demonstrated that *MMP9* expression levels had strong diagnostic values for both RA and control samples (GSE55325, AUC = 0.960; GSE77298, AUC = 0.938) (Fig. 8F). Interestingly, the KEGG analysis from the Enrichr database showed that *MMP9* is significantly enriched in the IL-17 signaling pathway and TNF signaling pathway (Fig. 9A). And we discovered two important pro-inflammatory cytokines, namely IL-6 and TNF-α, took part in IL-17 signaling pathway, as well as MMP9, which was associated with the TNF signaling pathway via KEGG mapping (Fig. 9B,C), which was further analyzed via ELISA and western blot.

### Sinomenine binds to MMP9, inhibits its expression, and the expression of inflammatory cytokines in LPS-induced RAW264.7 cells

**Figure 10:**
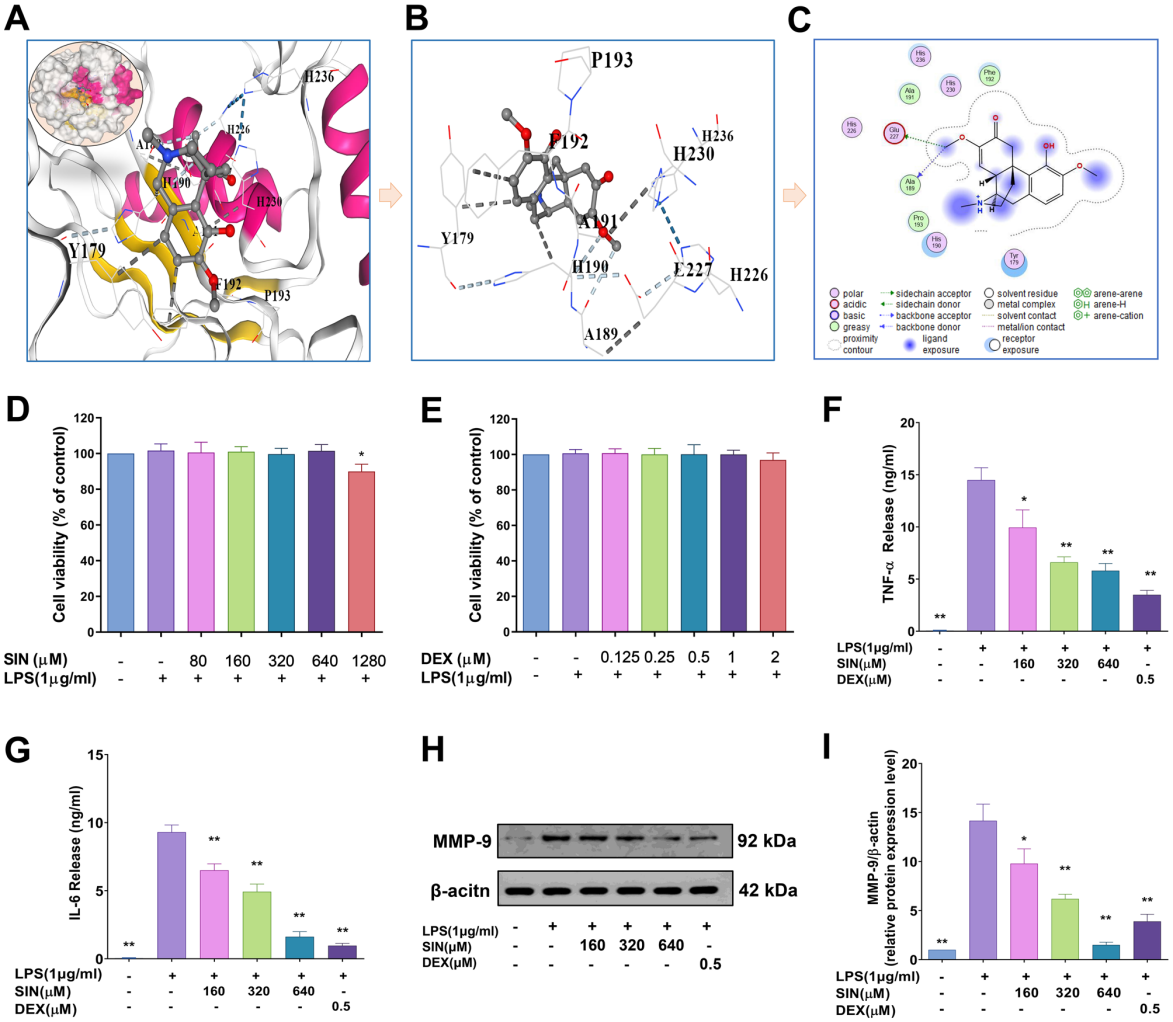
The therapeutic effect of Sinomenine (SIN) on LPS-induced RAW264.7 cells. (**A**–**C**) Representative three- and two-dimensional binding modes of MMP9 docking with SIN. (**D**) The action of SIN on RAW264.7 cells viability. (**E**) The action of DEX on the RAW264.7 cells viability. (**F**,**G**) The pharmacological action of SIN on TNF-α and IL-6 expression level in LPS-induced RAW264.7 cells. (**H**,**I**) Expression of MMP9 in-LPS induced RAW264.7 cells pretreated with SIN. Results are expressed as the mean value ± SD. *p < 0.05, **p < 0.01 indicates a significant difference from the model group (only Fig.10D indicates a significant difference from the control group).

The SIN for the treatment of RA was further investigated using molecular docking technology, ELISA, and Western blot. According to the results of the aforesaid bioinformatic study, *MMP9* was identified as a hub gene and was used for molecular docking with the SIN in CB-Dock2 platform, respectively. This study aimed to further investigate the binding affinity of MMP9 and SIN. The Vina score was calculated as an indication of binding affinity. The receptor-ligand docking hypothesis states that docking energy is inversely proportional to binding affinity, with lower docking energy indicating a greater binding affinity between the protein and the ligand. As presented in Table 2, CB-Dock2 output the top 5 conformations with the lowest binding energy value based on the calculation findings. The Vina score is connected to the binding capacity of the ligand and the receptor. The results showed that MMP9 exhibited a terrific Vina score of − 6.0 kcal/mol with SIN (Fig. 10A), and 2D illustration revealed that the stable binding of SIN to MMP9 was securely mediated through 4 amino acid residues, namely His190, Glu227, Ala189, and Phe192 (Fig. 10B,C). Our previous study showed a significant upregulation of MMP9 expression in RA patients, and SIN could dock with MMP9 with ideal results via molecular docking (Fig. 10A–C).

**Table 2:** The Binding affinity scores according to the CB-Dock2.

CurPocket ID	Cavity size volume (Å^3^)	Center (x, y, z)	Docking size (x, y, z)	Vina Score (kcal/mol)
C1	569	18, − 15, 21	19, 19, 28	− 6.0
C2	158	31, − 14, 20	19, 19, 19	− 6.0
C3	385	27, − 11, 4	19, 19, 19	− 5.9
C4	81	12, − 3, 24	19, 19, 19	− 5.8
C5	34	4, − 16, 17	19, 19, 19	− 5.5

To verify the accuracy of the previous prediction, we used LPS-induced RAW264.7 cells to prove some of the experimental results of our prediction. First, the CCK8 assay (Fig. 10D,E) was used to investigate whether SIN (with a concentration greater than 640 micromoles) has obvious toxic effects on cells, after which the concentration of SIN and positive drug (dexamethasone, DEX) with no obvious cytotoxicity (160–640 µM, 0.5 µM) were selected for follow-up experiments. Next, ELISA was performed to assess the expression of TNF-α and IL-6 in LPS-induced RAW264.7 cells (with or without SIN treatment). The results (Fig. 10F,G) showed that SIN inhibits the release of inflammatory factors (TNF-α and IL-6) in a dose-dependent manner. DEX also significantly inhibited the expression of TNF-α and IL-6 (Fig. 10F,G).

To study whether SIN can downregulate the overexpression of MMP9 protein, we evaluated the effect of SIN on the MMP9 protein in LPS-induced RAW264.7 cells. Western blot analysis was performed to assess the MMP9 protein expression in LPS-induced RAW264.7 cells (with or without SIN treatment). Remarkably, SIN inhibited the expression of MMP9 in a dose-dependent manner compared to the model group (Fig. 10H,I). Meanwhile, the positive drug (DEX) also significantly inhibited the expression of the MMP9 protein (Fig. 10H,I).

## Discussion

Rheumatoid arthritis (RA) is a systemic autoimmune disease characterized by extra-articular involvement and inflammatory arthritis^33^. Previous studies have suggested that infiltrating immune cells have an essential role in the development and progression of RA^34–37^. A variety of inflammatory cytokines (e.g., IL-1, IL-6, IL-8, and TNF-α) and matrix metalloproteinases (e.g., MMP-1, MMP-2, and MMP-9) that are released in synovial fluids by invading macrophages and are involved in T cell activation and proliferation as well as cell–cell contact mediation^12,29, 30^. The release of tissue-damaging enzymes results in the proliferation of inflammatory and joint destruction in RA^15,31^. As a result, it is critical to look for specific diagnostic markers and examine the infiltration patterns of RA immune cells to improve the prognosis of RA patients. In this study, we identified RA diagnostic biomarkers and assessed the influence of infiltrating immune cells inside RA.

We compared genes expressed in RA and control samples and then identified 4912 available DEGs for independent testing cohorts, including 2721 upregulation DEGs and 2191 downregulation DEGs. Following that, the DEGs were annotated using a function-related enrichment analysis. The GO-BP analysis indicated that these 2721 upregulated DEGs were tightly linked to immunological responses, macrophages, and inflaming signals (e.g., immune responses, inflammatory responses, innate immune responses, and macrophage activation regulation). As an outcome, we looked at genes associated with the quantity of tissue-infiltrated immune cells. We examined the number of 22 invading immune cell types in RA and control samples from both tests and cohorts using the CIBERSORT approach. Then, we further assessed the role of M1 macrophages in RA. Our findings revealed a notable distinction between RA and control samples in the GSE55457 and GSE12021 datasets, particularly emphasizing M1 macrophages alone. To explore the correlation between gene expression levels and the quantity of M1 macrophages, we constructed the WGCNA and identified gene modules associated with M1 macrophages. The WGCNA detected four hub gene modules and 1779 linked genes. According to Metascape, these chosen genes are mostly involved in immunological, inflammatory response, and cytokine synthesis, as well as positive immune response control. Consequently, SIN-related targets were identified using a public pharmacological target prediction database, and 226 target genes were obtained. Then, 24 common genes were chosen for further investigation. We discovered that these 24 overlapping genes were highly connected with the immune system in the Enrichr and Metascape databases.

To identify prospective diagnostic biomarkers for RA, we used 6 different cytoHubba algorithms on the aforementioned 24 overlapping genes and discovered 13 genes as probable candidate genes: *MMP9, IFNG, CDK1, CHEK1, BTK, PIK3CG, PIM2, CSF1R, AURKA, TLR8, PBK, AURKB,* and *TOP2A*. Then, a prediction model for the diagnosis of RA was created using the LASSO method to identify RA patients from healthy controls in the testing cohorts (MMP9 was not significant with a zero coefficient in the GSE100191 dataset). *MMP9* was screened with non-zero coefficients in the datasets GSE55325, GSE55457, and GSE77298, with a substantial difference in GSE55325 (p < 0.001) and GSE77298 (p < 0.001). Furthermore, the ROC curve's AUC value indicated its strong ability to distinguish RA samples from control samples. Furthermore, molecular docking studies revealed the SIN's ideal binding affinity to MMP9 protein, which leads to RA attenuation.

A number of studies showed that SIN has a substantial impact on RA treatment^38–42^. However, there is limited evidence that SIN can reduce MMP9 protein overexpression in the treatment of RA^20,37, 43^. RA cartilage is characterized by high levels of MMP9 in the synovial tissues and fluids, thereby contributing significantly to the degradation of connective tissue components^44^. Earlier investigations have proposed that Icariin, the active individual component of flavonoid glycosides derived from *Epimedium grandiflorum*, may alleviate RA by suppressing the expression of MMP9, a marker for osteoclasts, lowering Th17 cell counts, and impeding the activation of STAT3, thereby reducing the production of IL-17^45^. In this study, we found that SIN could significantly suppress the expression of MMP9 protein (Fig. 10H,I), which suggests that SIN could be used to treat patients with RA by inhibiting the overexpression of MMP9 protein. Consequently, this result provides more scientific research basis for SIN targeting MMP9 proteins to treat RA.

When activated, macrophages exhibit a role in inducing and exacerbating inflammation^46^. Their significance cannot be overstated during RA as they produce inflammation-boosting cytokines, fueling cartilage and bone destruction^12^. In various microenvironments, macrophages exhibit heterogeneity and can be differentiated into pro-inflammatory macrophages and anti-inflammatory macrophages, i.e., M1 and M2 macrophages, respectively. The interaction between activated M1 macrophages and Th1 cells promotes the synthesis of numerous inflammatory mediators in RA synovial tissue. These mediators include IL-6, TNF-α, and so on^47,48^.

Next, we selected M1 macrophages for further analysis and identified *MMP9* as a critical M1 macrophage-related gene, significantly enriched in the IL-17 and TNF signaling pathways. Furthermore, the therapeutic efficacy of SIN was validated in LPS-induced RAW264.7 cells line using ELISA to detect the content of TNF-α and IL-6. SIN significantly inhibited the production of TNF-α and IL-6 LPS-induced RAW264.7 cells (Fig. 10F,G).

In conclusion, various databases and new bioinformatics approaches were used to analyze the relevant targets of SIN treatment of RA for the first time, followed by the cell experiment preliminarily proving that SIN exerts its anti-inflammatory and anti-RA effects through the predicted target. We hope the current research results can provide some new scientific research ideas for the SIN treatment of RA.

## Conclusion

This work employed a variety of bioinformatics studies to provide important insights into the relationship between gene expression and the number of immune cells entering organs. Additionally, we identified MMP9 as a M1 macrophages biomarker related to the immune and inflammatory response in the development of RA. It further demonstrated that SIN presented an anti-inflammatory activity in LPS-stimulated RAW264.7 cells by inhibiting the MMP9 protein expression. Subsequently, this anti-inflammatory effect of SIN resulted in the suppression of inflammatory mediators namely TNF-α and IL-6. Additionally, further investigations should be conducted to evaluate the mechanisms through which SIN exhibits its anti-inflammatory effects using alternative cell lines and animal models.

### Supplementary Information

Supplementary Information 1.

Supplementary Information 2.

## Data Availability

Publicly available datasets (GSE12021, GSE55325, GSE55457, GSE77298, GSE93272, GSE77298, and GSE100191) were analyzed in this study. All the datasets presented in this study can be obtained from the GEO (http://www.ncbi.nlm.nih.gov/geo) database. Data is provided within the manuscript or supplementary information files and it is available upon request from the corresponding author.
