# Supplementary figures and images for: Sinomenine treats rheumatoid arthritis by inhibiting MMP9 and inflammatory cytokines expression: bioinformatics analysis and experimental validation
Source: Sci Rep. 2024 Jun 4;14:12786. doi: 10.1038/s41598-024-61769-x (PMC11151427; doi:10.1038/s41598-024-61769-x)

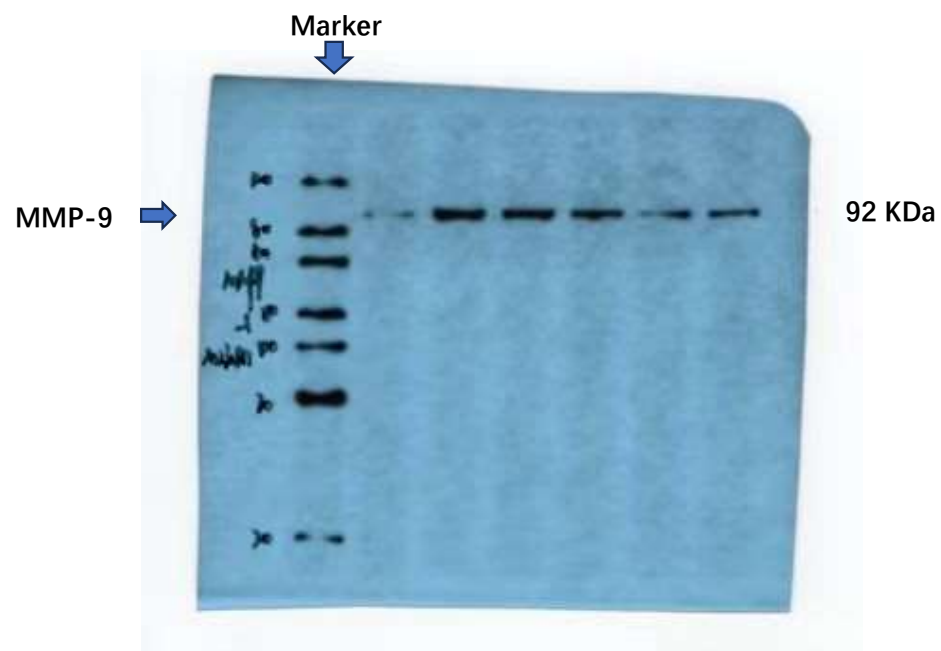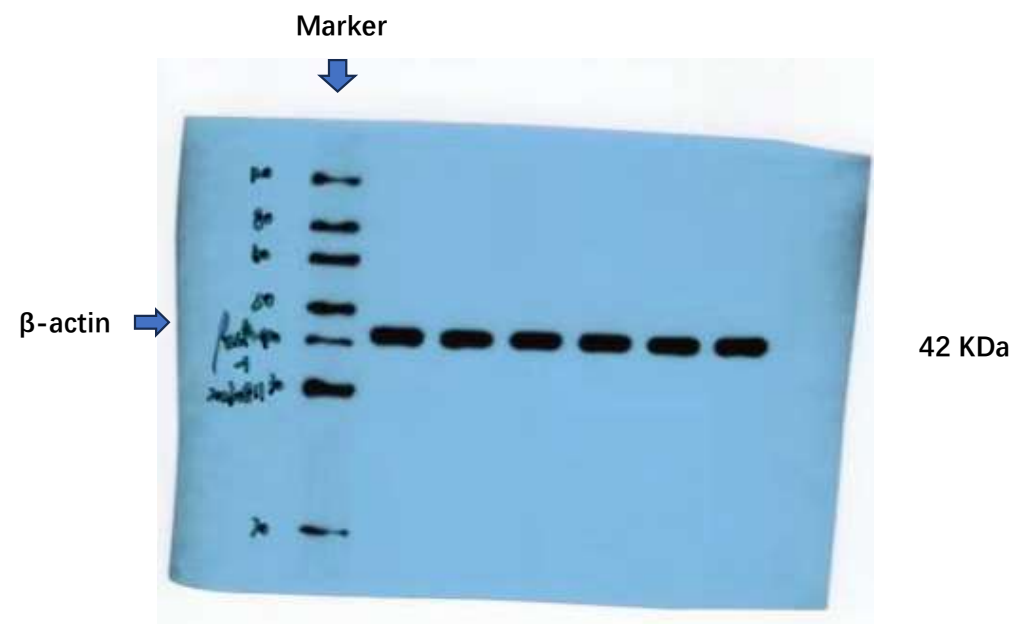

Supplement: Supplementary file 2 — Supplementary Information 2. [file 41598_2024_61769_MOESM2_ESM.pdf]
